# Supplementary material for: Bark beetle (Ips typographus) protein elicitors trigger temporally structured immune responses in Norway spruce (Picea abies) seedlings
Source: Front Plant Sci. 2026 Sep 8;17:1911806. doi: 10.3389/fpls.2026.1911806 (PMC13593760; doi:10.3389/fpls.2026.1911806)

A

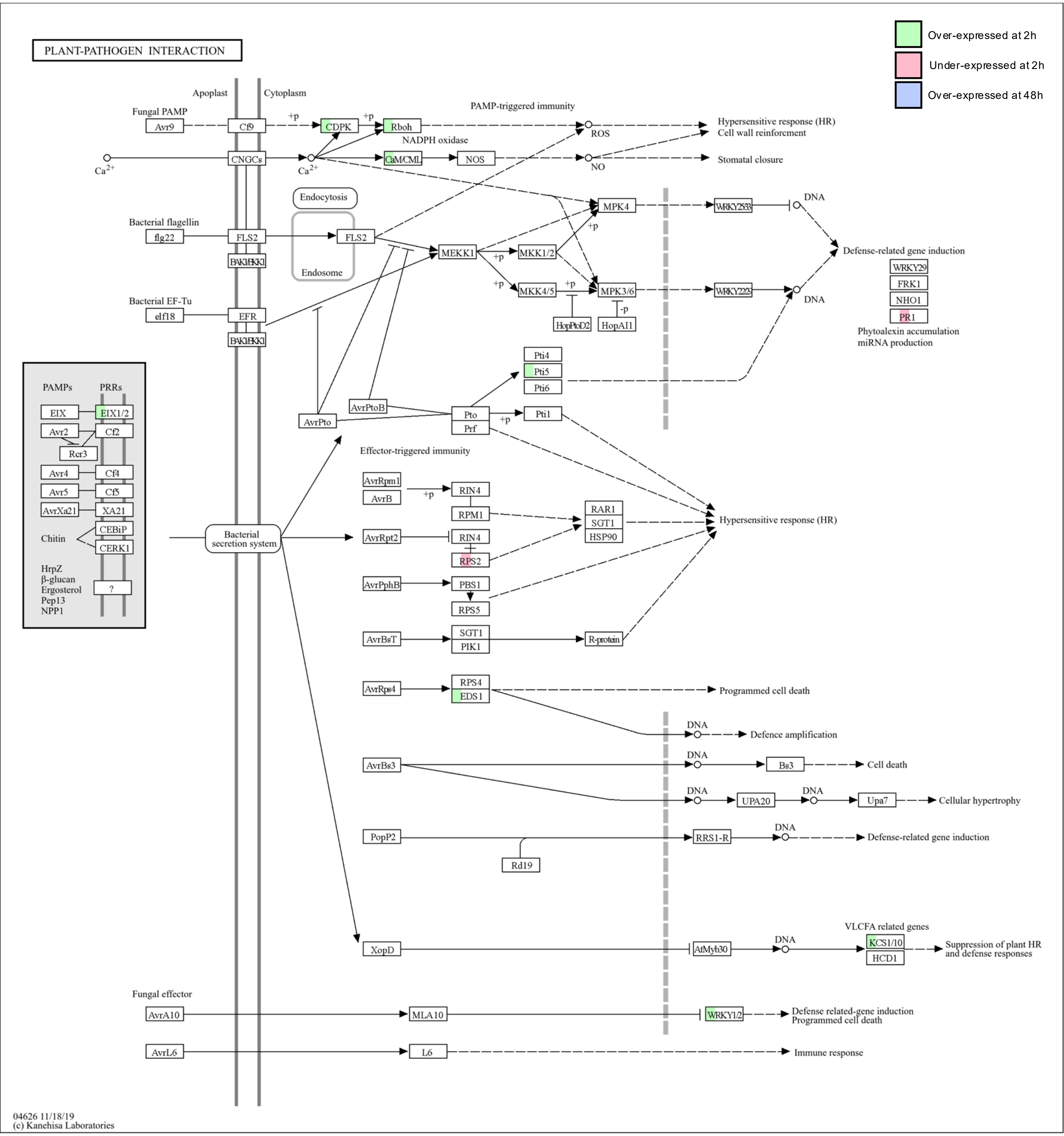
PLANT-PATHOGEN INTERACTION
Over-expressed at 2h
Under-expressed at 2h
Over-expressed at 48h
Apoplast
Cytoplasm
Fungal PAMP
Avr9
Cf9
+p
CDPK
+p
Rboh
NADPH oxidase
PAMP-triggered immunity
ROS
Hypersensitive response (HR)
Cell wall reinforcment
Ca2+
CNGCs
Ca2+
CaM/CML
NOS
NO
Stomatal closure
Endocytosis
Bacterial flagellin
flg22
FLS2
BAK1/BKK1
FLS2
Endosome
MEKK1
+p
MKK1/2
+p
MPK4
WRKY25/33
DNA
Defense-related gene induction
WRKY29
FRK1
NHO1
PR1
Phytoalexin accumulation
miRNA production
+p
MKK4/5
+p
MPK3/6
-p
WRKY22/29
DNA
HopPtoD2
HopAI1
Bacterial EF-Tu
elf18
EFR
BAK1/BKK1
PAMPs
PRRs
EIX
EIX1/2
Avr2
Cf2
Rcr3
Avr4
Cf4
Avr5
Cf5
AvrXa21
XA21
Chitin
CEBiP
CERK1
HrpZ
β-glucan
Ergosterol
Pep13
NPP1
?
Pti4
Pti5
Pti6
AvrPtoB
AvrPto
Pto
Prf
+p
Pti1
Effector-triggered immunity
Bacterial secretion system
AvrRpm1
AvrB
+p
RIN4
RPM1
RAR1
SGT1
HSP90
Hypersensitive response (HR)
AvrRpt2
RIN4
RPS2
AvrPphB
PBS1
RPS5
AvrBsT
SGT1
PIK1
R-protein
AvrRps4
RPS4
EDS1
Programmed cell death
DNA
Defence amplification
AvrBs3
DNA
Bs3
Cell death
DNA
UPA20
DNA
Upa7
Cellular hypertrophy
PopP2
RRS1-R
DNA
Defense-related gene induction
Rd19
VLCFA related genes
XopD
AtMyb30
DNA
KCS1/10
HCD1
Suppression of plant HR and defense responses
Fungal effector
AvrA10
MLA10
WRKY1/2
Defense related-gene induction
Programmed cell death
AvrL6
L6
Immune response
04626 11/18/19
(c) Kanehisa Laboratories

B

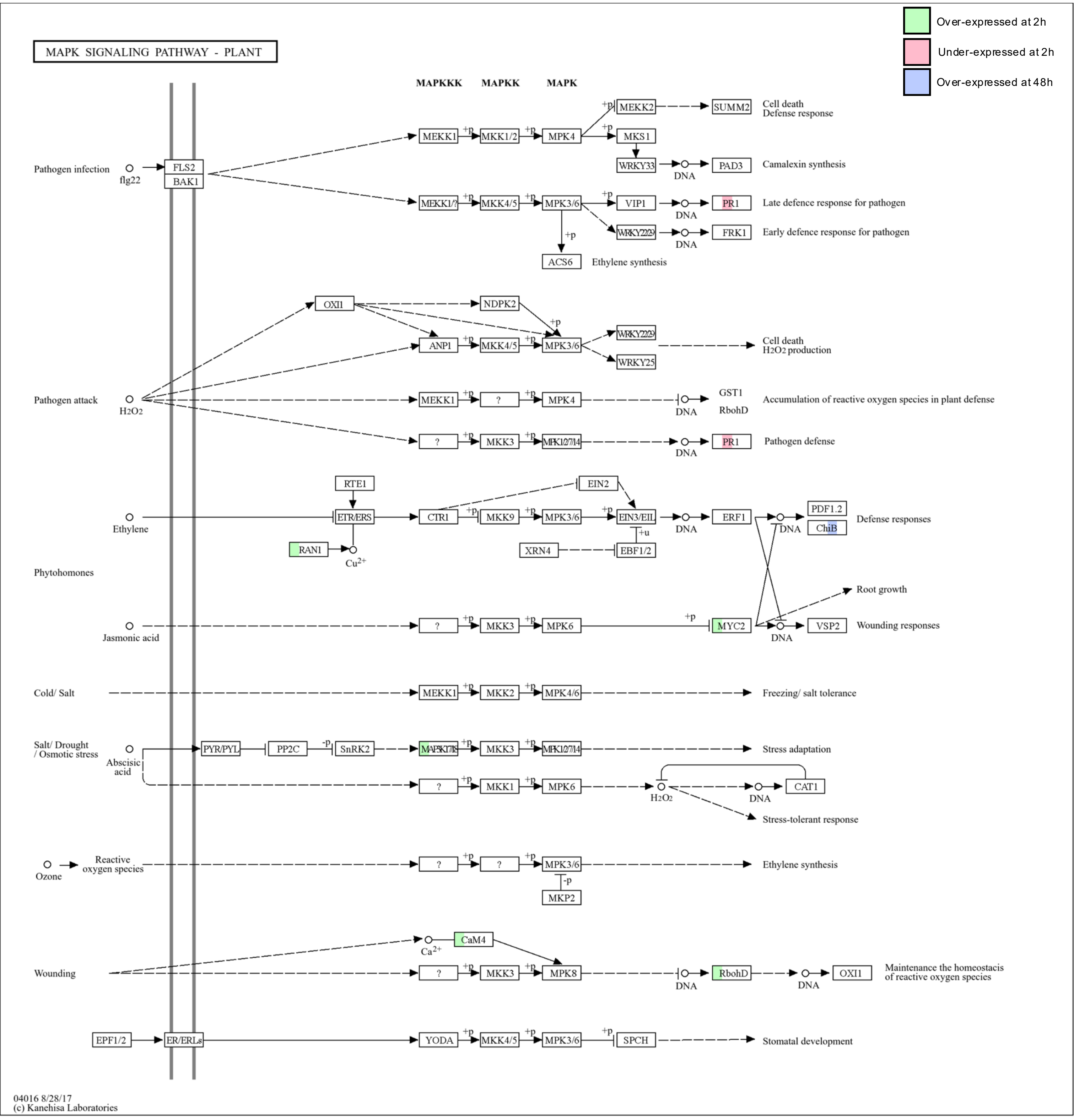

Over-expressed at 2h
Under-expressed at 2h
Over-expressed at 48h
MAPK SIGNALING PATHWAY - PLANT
MAPKKK
MAPKK
MAPK
Pathogen infection
flg22
FLS2
BAK1
MEKK1
MKK1/2
MPK4
MEKK2
SUMM2
Cell death
Defense response
MKS1
WRKY33
DNA
PAD3
Camalexin synthesis
MKK4/5
MPK3/6
VIP1
PR1
Late defence response for pathogen
FRK1
Early defence response for pathogen
ACS6
Ethylene synthesis
OXI1
NDPK2
ANP1
WRKY25
Cell death
H2O2 production
Pathogen attack
H2O2
GST1
RbohD
Accumulation of reactive oxygen species in plant defense
MKK3
Pathogen defense
RTE1
EIN2
Ethylene
ETR/ERS
CTR1
MKK9
EIN3/EIL
ERF1
PDF1.2
ChiB
Defense responses
RAN1
Cu2+
XRN4
EBF1/2
Phytohomones
Root growth
Jasmonic acid
MPK6
MYC2
VSP2
Wounding responses
Cold/ Salt
MKK2
MPK4/6
Freezing/ salt tolerance
Salt/ Drought
/ Osmotic stress
Abscisic acid
PYR/PYL
PP2C
SnRK2
MKK3
Stress adaptation
MKK1
CAT1
Stress-tolerant response
Ozone
Reactive oxygen species
Ethylene synthesis
MKP2
Ca2+
CaM4
Wounding
MPK8
OXI1
Maintenance the homeostacis
of reactive oxygen species
EPF1/2
ER/ERLs
YODA
SPCH
Stomatal development
04016 8/28/17
(c) Kanehisa Laboratories

C

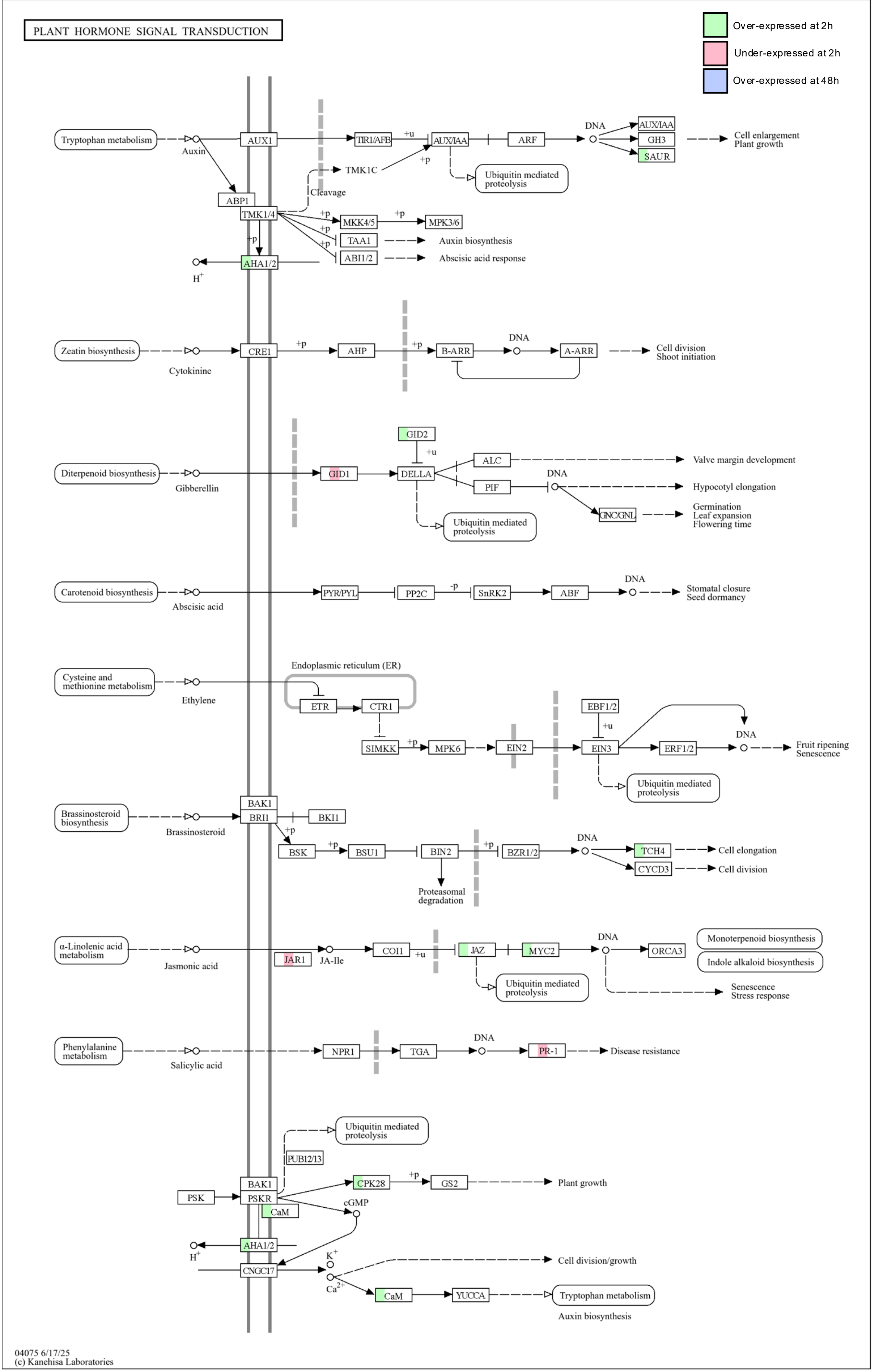

PLANT HORMONE SIGNAL TRANSDUCTION
Over-expressed at 2h
Under-expressed at 2h
Over-expressed at 48h
Tryptophan metabolism
Auxin
AUX1
TIR1/AFB
+u
AUX/IAA
ARF
DNA
AUX/IAA
GH3
SAUR
Cell enlargement
Plant growth
TMK1C
+p
Cleavage
Ubiquitin mediated proteolysis
ABP1
TMK1/4
+p
MKK4/5
MPK3/6
TAA1
Auxin biosynthesis
ABI1/2
Abscisic acid response
AHA1/2
H+
Zeatin biosynthesis
Cytokinine
CRE1
AHP
B-ARR
A-ARR
Cell division
Shoot initiation
GID2
+u
Diterpenoid biosynthesis
Gibberellin
GID1
DELLA
ALC
Valve margin development
PIF
Hypocotyl elongation
GNC/GNL
Germination
Leaf expansion
Flowering time
Carotenoid biosynthesis
Abscisic acid
PYR/PYL
PP2C
-p
SnRK2
ABF
Stomatal closure
Seed dormancy
Endoplasmic reticulum (ER)
Cysteine and methionine metabolism
Ethylene
ETR
CTR1
EBF1/2
SIMKK
MPK6
EIN2
EIN3
ERF1/2
Fruit ripening
Senescence
Brassinosteroid biosynthesis
Brassinosteroid
BAK1
BRI1
BKI1
BSK
BSU1
BIN2
BZR1/2
TCH4
Cell elongation
CYCD3
Cell division
Proteasomal degradation
α-Linolenic acid metabolism
Jasmonic acid
JAR1
JA-Ile
COI1
JAZ
MYC2
ORCA3
Monoterpenoid biosynthesis
Indole alkaloid biosynthesis
Senescence
Stress response
Phenylalanine metabolism
Salicylic acid
NPR1
TGA
PR-1
Disease resistance
PUB12/13
PSK
PSKR
CPK28
GS2
Plant growth
CaM
cGMP
K+
CNGC17
Cell division/growth
Ca2+
YUCCA
Tryptophan metabolism
Auxin biosynthesis
04075 6/17/25
(c) Kanehisa Laboratories

D

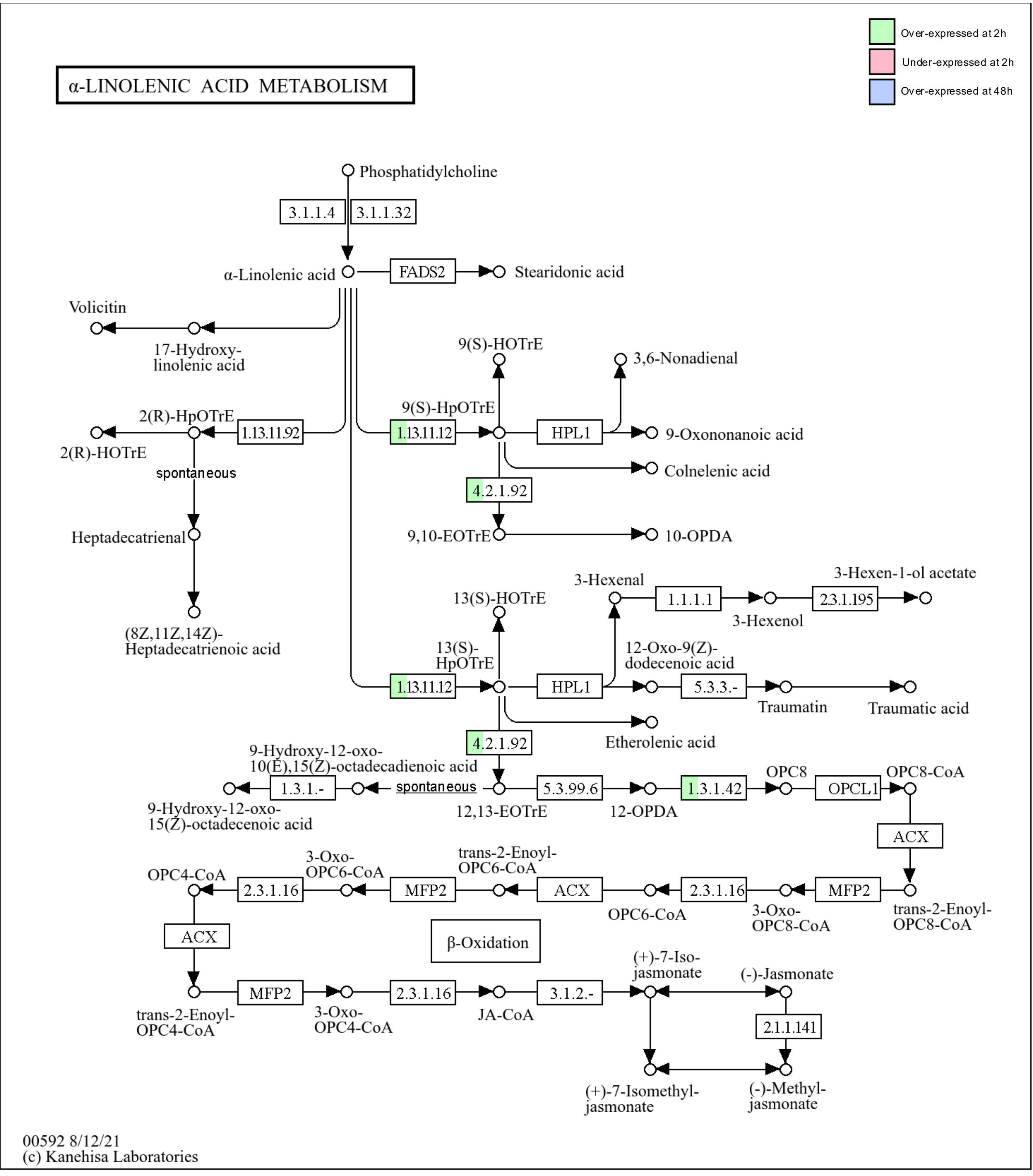
Over-expressed at 2h
Under-expressed at 2h
Over-expressed at 48h
α-LINOLENIC ACID METABOLISM
Phosphatidylcholine
3.1.1.4
3.1.1.32
α-Linolenic acid
FADS2
Stearidonic acid
Volicitin
17-Hydroxy-linolenic acid
9(S)-HOTrE
3,6-Nonadienal
2(R)-HpOTrE
1.13.11.92
2(R)-HOTrE
9(S)-HpOTrE
1.13.11.12
HPL1
9-Oxononanoic acid
Colnelenic acid
spontaneous
4.2.1.92
Heptadecatrienal
9,10-EOTrE
10-OPDA
(8Z,11Z,14Z)-Heptadecatrienoic acid
13(S)-HOTrE
3-Hexenal
1.1.1.1
3-Hexenol
3-Hexen-1-ol acetate
2.3.1.195
13(S)-HpOTrE
12-Oxo-9(Z)-dodecenoic acid
5.3.3.-
Traumatin
Traumatic acid
Etherolenic acid
9-Hydroxy-12-oxo-10(E),15(Z)-octadecadienoic acid
1.3.1.-
5.3.99.6
1.3.1.42
OPC8
OPCL1
OPC8-CoA
9-Hydroxy-12-oxo-15(Z)-octadecenoic acid
12,13-EOTrE
12-OPDA
ACX
OPC4-CoA
2.3.1.16
3-Oxo-OPC6-CoA
MFP2
trans-2-Enoyl-OPC6-CoA
OPC6-CoA
3-Oxo-OPC8-CoA
trans-2-Enoyl-OPC8-CoA
β-Oxidation
(+)-7-Iso-jasmonate
(-)-Jasmonate
trans-2-Enoyl-OPC4-CoA
3-Oxo-OPC4-CoA
JA-CoA
3.1.2.-
2.1.1.141
(+)-7-Isomethyl-jasmonate
(-)-Methyl-jasmonate
00592 8/12/21
(c) Kanehisa Laboratories

E

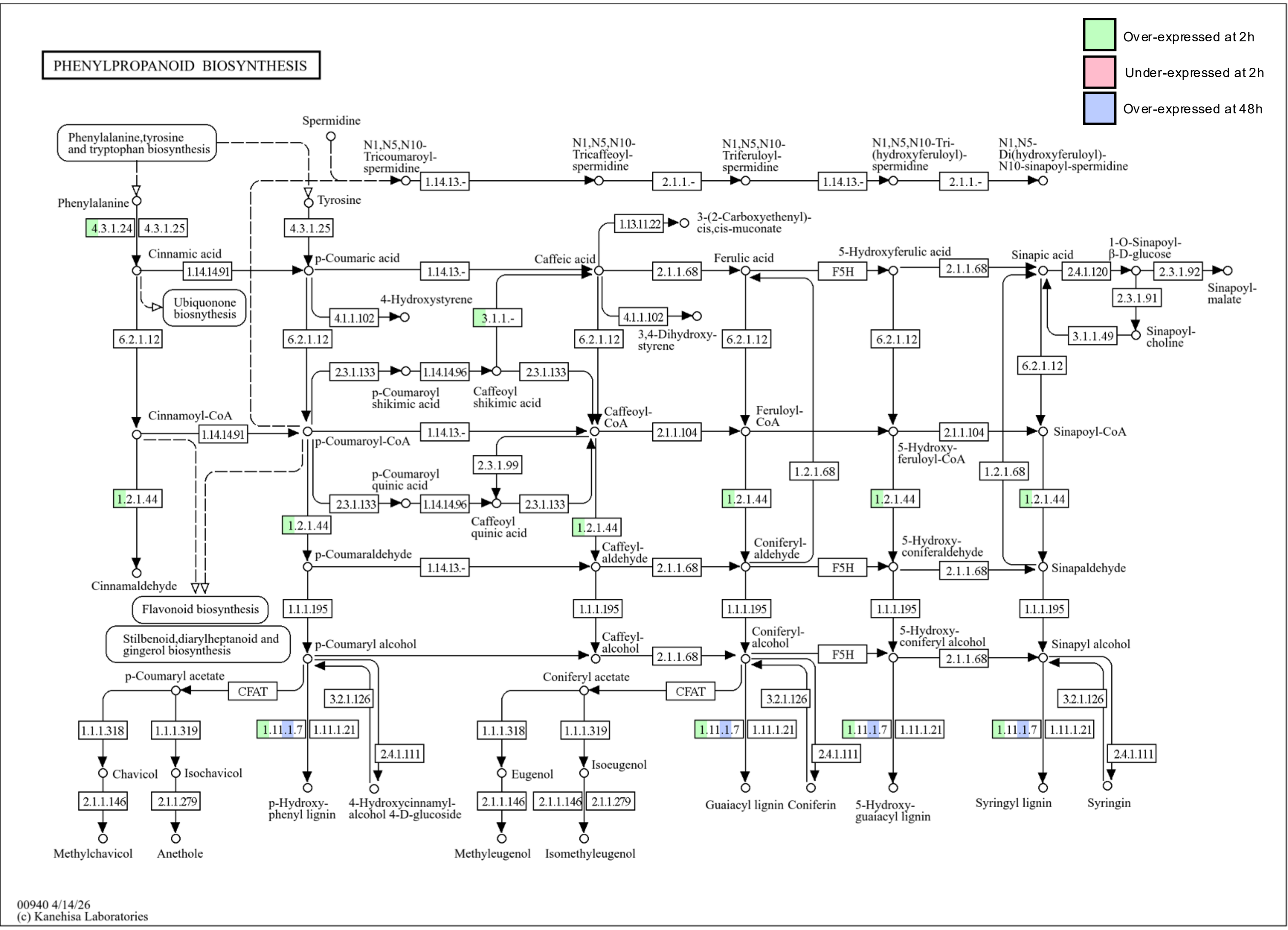

PHENYLPROPANOID BIOSYNTHESIS
Over-expressed at 2h
Under-expressed at 2h
Over-expressed at 48h
Phenylalanine,tyrosine and tryptophan biosynthesis
Spermidine
N1,N5,N10-Tricoumaroyl-spermidine
1.14.13.-
N1,N5,N10-Tricaffeoyl-spermidine
2.1.1.-
N1,N5,N10-Triferuloyl-spermidine
1.14.13.-
N1,N5,N10-Tri-(hydroxyferuloyl)-spermidine
2.1.1.-
N1,N5-Di(hydroxyferuloyl)-N10-sinapoyl-spermidine
Phenylalanine
Tyrosine
4.3.1.24
4.3.1.25
4.3.1.25
1.13.11.22
3-(2-Carboxyethenyl)-cis,cis-muconate
Cinnamic acid
1.14.14.91
p-Coumaric acid
1.14.13.-
Caffeic acid
2.1.1.68
Ferulic acid
F5H
5-Hydroxyferulic acid
2.1.1.68
Sinapic acid
2.4.1.120
1-O-Sinapoyl-β-D-glucose
2.3.1.92
Sinapoyl-malate
Ubiquonone biosynthesis
4.1.1.102
4-Hydroxystyrene
3.1.1.-
4.1.1.102
3,4-Dihydroxy-styrene
2.3.1.91
3.1.1.49
Sinapoyl-choline
6.2.1.12
6.2.1.12
6.2.1.12
6.2.1.12
6.2.1.12
6.2.1.12
2.3.1.133
1.14.14.96
2.3.1.133
p-Coumaroyl shikimic acid
Caffeoyl shikimic acid
Cinnamoyl-CoA
1.14.14.91
p-Coumaroyl-CoA
1.14.13.-
Caffeoyl-CoA
2.1.1.104
Feruloyl-CoA
5-Hydroxy-feruloyl-CoA
2.1.1.104
Sinapoyl-CoA
2.3.1.99
p-Coumaroyl quinic acid
2.3.1.133
1.14.14.96
2.3.1.133
Caffeoyl quinic acid
1.2.1.68
1.2.1.68
1.2.1.44
1.2.1.44
1.2.1.44
1.2.1.44
1.2.1.44
1.2.1.44
p-Coumaraldehyde
1.14.13.-
Caffeyl-aldehyde
2.1.1.68
Coniferyl-aldehyde
F5H
5-Hydroxy-coniferaldehyde
2.1.1.68
Sinapaldehyde
Cinnamaldehyde
Flavonoid biosynthesis
Stilbenoid,diarylheptanoid and gingerol biosynthesis
1.1.1.195
1.1.1.195
1.1.1.195
1.1.1.195
1.1.1.195
p-Coumaryl alcohol
Caffeyl-alcohol
2.1.1.68
Coniferyl-alcohol
F5H
5-Hydroxy-coniferyl alcohol
2.1.1.68
Sinapyl alcohol
p-Coumaryl acetate
CFAT
Coniferyl acetate
CFAT
3.2.1.126
3.2.1.126
3.2.1.126
1.1.1.318
1.1.1.319
1.11.1.7
1.11.1.21
1.1.1.318
1.1.1.319
1.11.1.7
1.11.1.21
1.11.1.7
1.11.1.21
1.11.1.7
1.11.1.21
2.4.1.111
2.4.1.111
2.4.1.111
Chavicol
Isochavicol
Eugenol
Isoeugenol
2.1.1.146
2.1.1.279
2.1.1.146
2.1.1.146
2.1.1.279
p-Hydroxy-phenyl lignin
4-Hydroxycinnamyl-alcohol 4-D-glucoside
Guaiacyl lignin
Coniferin
5-Hydroxy-guaiacyl lignin
Syringyl lignin
Syringin
Methylchavicol
Anethole
Methyleugenol
Isomethyleugenol
00940 4/14/26
(c) Kanehisa Laboratories

F

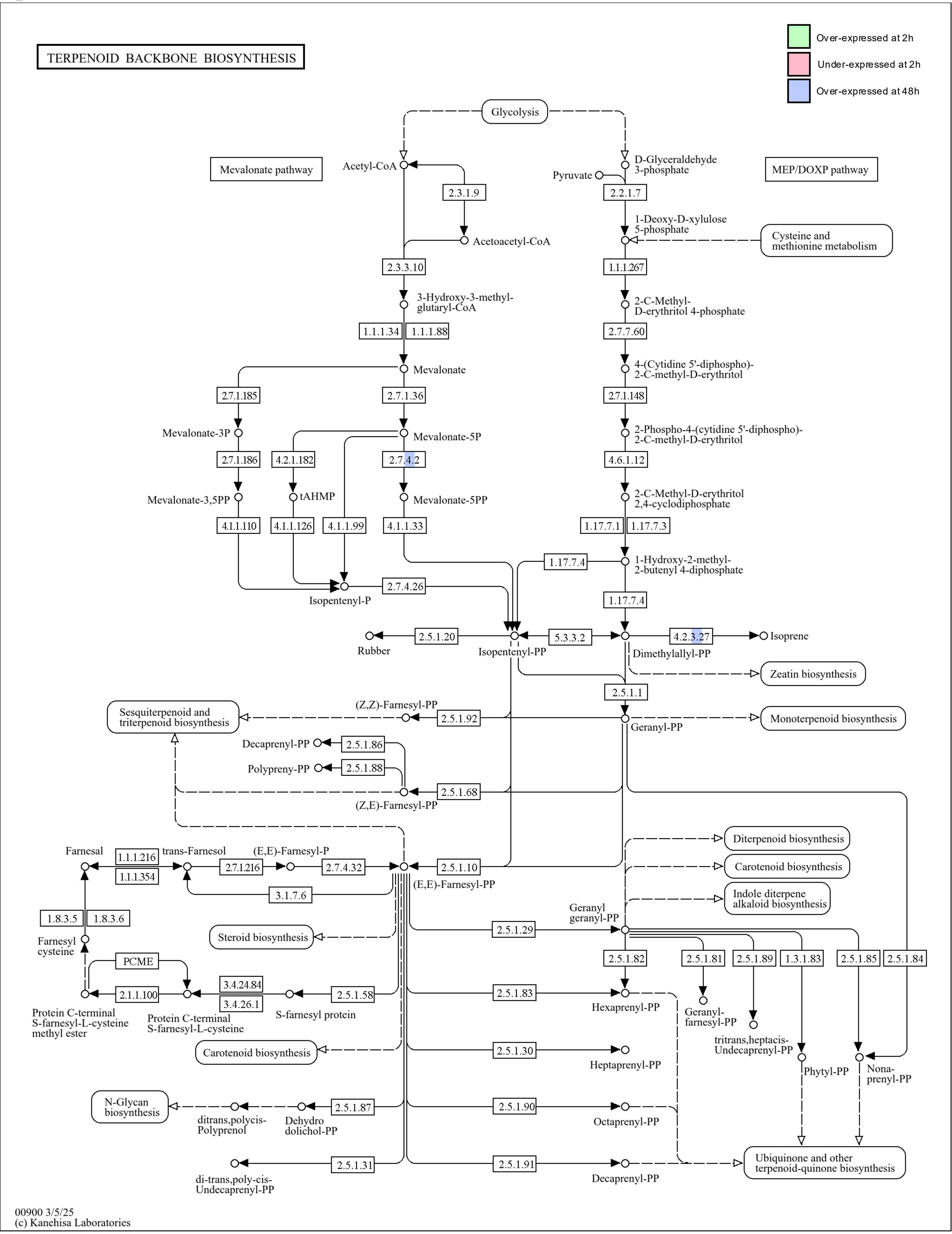
TERPENOID BACKBONE BIOSYNTHESIS
Over-expressed at 2h
Under-expressed at 2h
Over-expressed at 48h
Glycolysis
Mevalonate pathway
MEP/DOXP pathway
Acetyl-CoA
2.3.1.9
Acetoacetyl-CoA
2.3.3.10
3-Hydroxy-3-methyl-glutaryl-CoA
1.1.1.34
1.1.1.88
Mevalonate
2.7.1.185
2.7.1.36
Mevalonate-3P
Mevalonate-5P
2.7.1.186
4.2.1.182
2.7.4.2
Mevalonate-3,5PP
tAHMP
Mevalonate-5PP
4.1.1.110
4.1.1.126
4.1.1.99
4.1.1.33
Isopentenyl-P
2.7.4.26
D-Glyceraldehyde 3-phosphate
Pyruvate
2.2.1.7
1-Deoxy-D-xylulose 5-phosphate
Cysteine and methionine metabolism
1.1.1.267
2-C-Methyl-D-erythritol 4-phosphate
2.7.7.60
4-(Cytidine 5'-diphospho)-2-C-methyl-D-erythritol
2.7.1.148
2-Phospho-4-(cytidine 5'-diphospho)-2-C-methyl-D-erythritol
4.6.1.12
2-C-Methyl-D-erythritol 2,4-cyclodiphosphate
1.17.7.1
1.17.7.3
1.17.7.4
1-Hydroxy-2-methyl-2-butenyl 4-diphosphate
1.17.7.4
Rubber
2.5.1.20
Isopentenyl-PP
5.3.3.2
Dimethylallyl-PP
4.2.3.27
Isoprene
Zeatin biosynthesis
2.5.1.1
Sesquiterpenoid and triterpenoid biosynthesis
(Z,Z)-Farnesyl-PP
2.5.1.92
Geranyl-PP
Monoterpenoid biosynthesis
Decaprenyl-PP
2.5.1.86
Polypreny-PP
2.5.1.88
(Z,E)-Farnesyl-PP
2.5.1.68
Farnesal
1.1.1.216
1.1.1.354
trans-Farnesol
(E,E)-Farnesyl-P
2.7.1.216
2.7.4.32
(E,E)-Farnesyl-PP
2.5.1.10
3.1.7.6
Diterpenoid biosynthesis
Carotenoid biosynthesis
Indole diterpene alkaloid biosynthesis
Geranyl geranyl-PP
1.8.3.5
1.8.3.6
Farnesyl cysteine
Steroid biosynthesis
2.5.1.29
PCME
3.4.24.84
3.4.26.1
2.1.1.100
2.5.1.58
Protein C-terminal S-farnesyl-L-cysteine methyl ester
Protein C-terminal S-farnesyl-L-cysteine
S-farnesyl protein
2.5.1.82
2.5.1.81
2.5.1.89
1.3.1.83
2.5.1.85
2.5.1.84
2.5.1.83
Hexaprenyl-PP
Geranyl-farnesyl-PP
tritrans,heptacis-Undecaprenyl-PP
Phytyl-PP
Nona-prenyl-PP
Carotenoid biosynthesis
2.5.1.30
Heptaprenyl-PP
N-Glycan biosynthesis
ditrans,polycis-Polyprenol
Dehydro dolichol-PP
2.5.1.87
2.5.1.90
Octaprenyl-PP
Ubiquinone and other terpenoid-quinone biosynthesis
di-trans,poly-cis-Undecaprenyl-PP
2.5.1.31
2.5.1.91
Decaprenyl-PP
00900 3/5/25
(c) Kanehisa Laboratories

MA_107643g0010

G

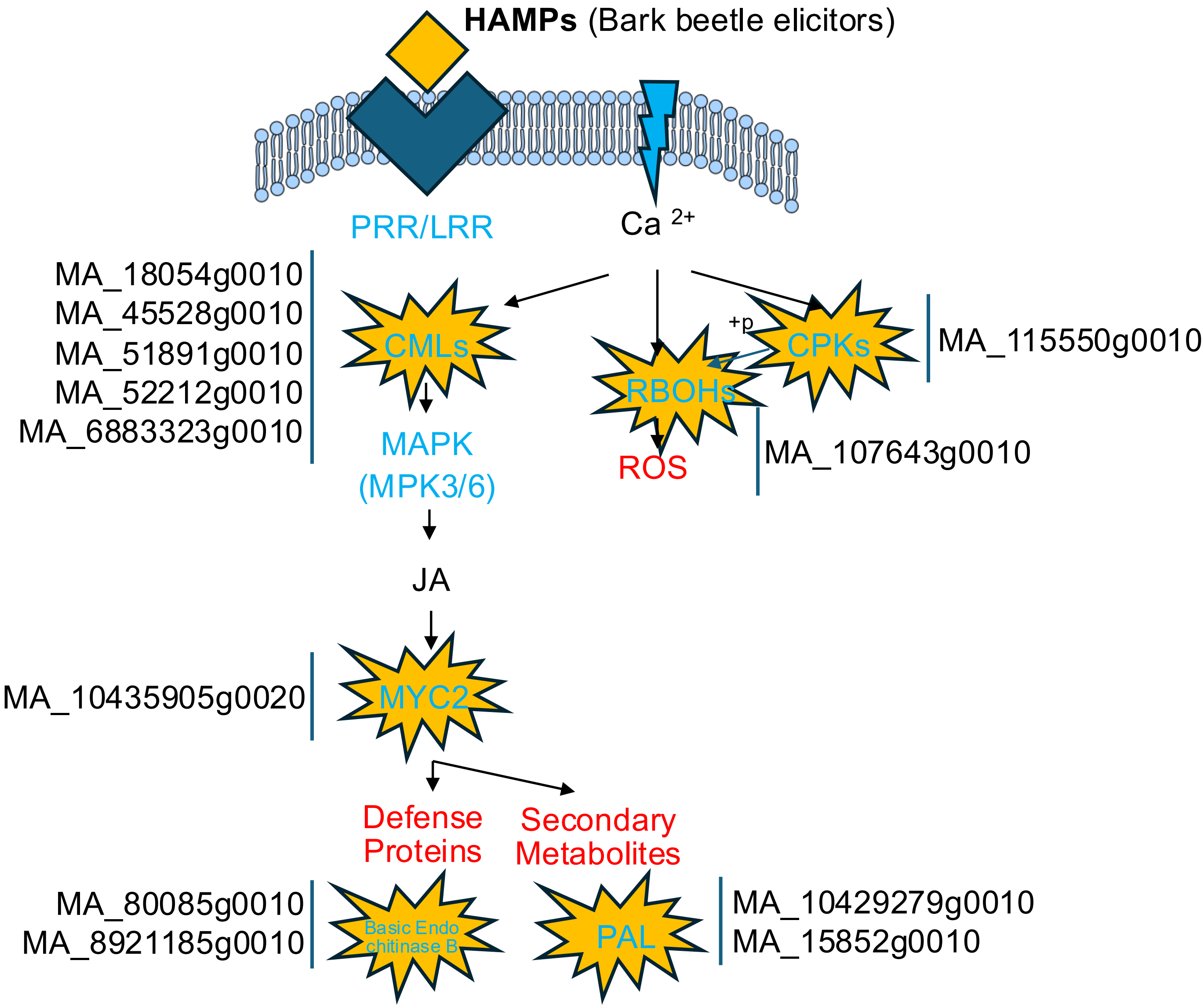

Supplement: Supplementary file 1 [file DataSheet1.pdf]
